# An Investigation into the Relationship Between Soft Tissue Body Composition and Bone Mineral Density in a Young Adult Twin Sample

**DOI:** 10.1002/jbmr.192

**Published:** 2010-07-23

**Authors:** Leonie H Bogl, Antti Latvala, Jaakko Kaprio, Olli Sovijärvi, Aila Rissanen, Kirsi H Pietiläinen

**Affiliations:** 1 The Finnish Twin Cohort Study, Department of Public Health, University of HelsinkiHelsinki, Finland; 2 Department of Mental Health and Substance Abuse Services, National Institute for Health and WelfareHelsinki, Finland; 3 Institute for Molecular MedicineHelsinki, Finland; 4 Obesity Research Unit, Department of Psychiatry, Helsinki University Central HospitalHelsinki, Finland; 5 Department of Medicine, Helsinki University Central HospitalHelsinki, Finland

**Keywords:** BONE MINERAL DENSITY, FAT MASS, LEAN MASS, TWIN STUDIES, GENETIC CORRELATION

## Abstract

The purpose of this study was to investigate the relationship of fat mass (FM) and lean mass (LM) with bone mineral density (BMD) independent of genetic effects. We also assessed the extent to which genetic and environmental influences explain the associations between these phenotypes. Body composition and BMD were measured using dual-energy X-ray absorptiometry in 57 monozygotic and 92 same-sex dizygotic twin pairs, aged 23 to 31 years, chosen to represent a wide range of intrapair differences in body mass index (BMI; 0 to 15.2 kg/m^2^). Heritability estimates were adjusted for height and gender. In multiple linear regression analysis, intrapair differences in both FM and LM were independently associated with intrapair differences in BMD at most skeletal sites after adjustment for gender and differences in height. Within monozygotic and dizygotic pairs, LM was a significantly stronger predictor of whole-body BMD than FM (*p* < .01). Additive genetic factors explained 87% [95% confidence interval (CI) 80%–91%), 81% (95% CI 70%–88%), and 61% (95% CI 41%–75%) of the variation in whole-body BMD, LM, and FM, respectively. Additive genetic factors also accounted for 69% to 88% of the covariance between LM and BMD and for 42% to 72% of the covariance between FM and BMD depending on the skeletal site. The genetic correlation between LM and whole-body BMD (*r*_*g*_ = 0.46, 95% CI 0.32–0.58) was greater than that of FM and whole-body BMD (*r*_*g*_ = 0.25, 95% CI 0.05–0.42). In conclusion, our data indicate that peak BMD is influenced by acquired body weight as well as genetic factors. In young adulthood, LM and BMD may have more genes in common than do FM and BMD. © 2011 American Society for Bone and Mineral Research.

## Introduction

The two complex diseases obesity and osteoporosis are both growing global public health problems.(1,2) Although studied extensively, the interrelationship between these two conditions is poorly understood. Increased body weight and body mass index (BMI) are associated with increased bone mineral density (BMD) in most studies(3–5); however, the opposite also has been reported in extremely obese (percent body fat > 55%) postmenopausal women(6) and obese women with Prader-Willi syndrome.(7) Controversy continues to surround the topic of whether fat mass (FM) is associated with BMD after adjusting for lean mass (LM) at different ages.(4,8–11) Potential mechanisms by which soft tissue and BMD could be associated include the effect of soft tissue mass on skeletal loading and the association of FM with the secretion of bone-active hormones from the pancreatic beta cells and the adipocytes.(12)

One explanation for the inconsistent results of several previous studies might be that the relationship between FM and BMD is subject to confounding.(13) Possible confounding factors in the fat-bone relationship include, among others, diet, physical activity, and socioeconomic status. Confounding also may arise from unknown and unmeasured factors, such as shared (environmental and/or genetic) factors that predispose to obesity as well as high BMD. It is well known that FM, BMI, and BMD are all under strong genetic control(14–16); thus a common set of genes may influence both obesity and BMD. For instance, polymorphisms in the *interleukin 6 receptor* gene were found to have effects on both BMI and BMD in a study of postmenopausal Spanish women.(17)

Most previous studies examining the association between body composition and bone mass were unable to tease apart the effect of genes and environment. Twin studies, however, can provide a very powerful model to study effects of acquired body weight.(18–20) To date, only a few twin studies have attempted to assess the relationship between soft tissue composition and BMD independent of genetic influences.(15,21) To our knowledge, only one study has used a quantitative genetic approach to explore whether the well-known relationship between body weight and BMD is in fact due to shared genetic factors. In that sample of 57 monozygotic (MZ) and 55 dizygotic (DZ) female twin pairs with a mean age of 53 years, the relationship between FM with BMD and between LM with BMD was mediated mainly via common environmental influences.(15) It is not known, however, whether these results hold for younger adults.

Thus our aim was to use the cotwin control design to examine the associations between soft tissue body composition and BMD at different skeletal sites, controlling for genetic factors. In addition, we used quantitative genetic analyses to examine whether the associations among LM, FM, and BMD can be explained by shared genetic, common, or unique environmental factors in young adulthood.

## Methods and Procedures

### Subjects and study design

The subjects of this study were a subset of the population-based longitudinal FinnTwin16 study, which consists of virtually all twins born between 1975 and 1979.(22) In FinnTwin16, height and weight have been reported as part of a questionnaire on health and behavioral habits at 16, 17, 18.5, and 22-27 years of age. The response rates were high (83% to 97%) in each survey. Self-reported weight and height at the last follow-up questionnaire were used to identify twin pairs with a wide range of intrapair differences in BMI for this study, in which the twins were measured clinically at the study center. The sample of 304 twin individuals included 20 monozygotic and 53 dizygotic pairs extremely discordant for BMI (intrapair difference > 3 kg/m^2^) and 18 monozygotic and 13 dizygotic pairs concordant for BMI (intrapair difference < 1 kg/m^2^) (EDAC = extremely discordant and concordant). Thus 21 monozygotic and 26 dizygotic had intrapair differences in BMI ranging from 1 to 3 kg/m^2^. For two male subjects, data were available for only one member of the twin pair. Of the total of 304 twin individuals, two individuals (one monozygotic female twin pair) were excluded because they did not fit within the dual-energy X-ray absorptiometry (DXA) scanner image zone, and one female individual was excluded owing to missing data, leaving a total of 301 subjects (28 male monozygotic pairs, 29 female monozygotic pairs, 48 male dizygotic pairs, 44 female dizygotic pairs, 2 male and 1 female twin individuals). Body composition and BMD were measured using DXA(23) (software Version 8.8, Lunar Prodigy, Madison, WI, USA). The measurements were carried out at a single clinical center. Bone mass measurements were made for the head, arms, ribs, legs, pelvis, spine, and whole body. DXA is a three-compartment model, dividing the body into total-body mineral mass, FM, and LM, the latter being the remaining bone-free, fat-free tissue mass.(23,24) Height and weight were measured barefoot and wearing underwear. Height was measured to the nearest 0.5 cm and weight to the nearest 0.1 kg. Zygosity was confirmed by genotyping of 10 informative genetic markers.(25) All subjects signed an informed consent, and the Ethical Committee of the Helsinki University Hospital approved this study.

### Statistical analyses

The basic statistical analyses were performed using the Stata statistical software (Release 9.0, StataCorp., College Station, TX, USA). The normality of the variables was assessed by the Shapiro-Wilk test. Height-adjusted Pearson's partial correlation coefficients and linear regression for survey data were calculated to determine the associations between anthropometric variables and BMD in individual twins. In these analyses, non–normally distributed data were used after logarithmic transformation, and clustering of correlated observations from twin pairs was controlled for when computing standard errors of the coefficients using robust estimators of variance.(26) Correlation analysis on the combined group of men and women additionally were adjusted for gender. The Wald test (*t* test adapted for clustered twin data) for independent samples was used to compare males and females and monozygotic and dizygotic twins. Pearson and Spearman partial correlation coefficients adjusted for height were used to analyze relationships between intrapair differences in anthropometric variables and intrapair differences in BMD depending on the distribution patterns of the variables. The independent relationship of soft tissue composition with BMD was evaluated by multiple linear regression analysis, controlling for height and gender. Monozygotic twins are genetically identical, and they also share much of their rearing environment during childhood and adolescence. Thus, if a positive association between body weight and BMD is found in individual-level analyses or within dizygotic pairs but not within monozygotic pairs, it is likely that the association is due to shared genetic factors affecting both weight and BMD. However, if the association is also seen within monozygotic pairs, the increase in BMD is a direct consequence of the acquired body weight. Intraclass correlations adjusted for gender and height were calculated to measure the similarity of monozygotic and dizygotic twins and to provide evidence for the presence of genetic effects. *p* Values of less than .05 were considered significant.

### Quantitative genetic analysis

Classic twin modeling is based on the fact that dizygotic twins, like nontwin full siblings, share on average 50% of their segregating genes, whereas monozygotic twins are genetically identical. The genetic variation can be divided into additive (*A*) and dominant (*D*) genetic effects, which have an expected correlation of 1 for both within monozygotic pairs and correlations of 0.5 and 0.25, respectively, within dizygotic pairs. *A* refers to the sum of the allelic effects on the phenotype over all susceptible loci, whereas *D* refers to interaction effects between alleles at the same locus. The environmental variation can be divided into common (*C*) and unique environmental (*E*) effects, which have (by definition) a correlation of 1 and 0, respectively, within both monozygotic and dizygotic twin pairs. The common environment includes all environmental factors that make the twin pair similar for the trait, such as shared childhood experiences, parental socioeconomic status, and shared friends and peers. The unique environment includes all environmental factors and experiences that make siblings in the family dissimilar, such as diseases or accidents that have affected only one sibling within a pair. The *E* component also includes measurement error because this is a random effect not correlated between twins.(27) It is possible to fit models based on different combinations of these parameters: *ADE*, *ACE*, *AE*, *CE*, and *E*; but effects owing to dominance and common environmental effects cannot be estimated simultaneously with data limited to those from twins reared together.(27) The classic method of analyzing twin data assumes the absence of gene-gene and gene-environment interactions. Further, the twin model assumes that there is random mating with respect to the traits in question. Positive assortative mating would increase the dizygotic but not the monozygotic correlations and thus inflate the estimates of common environmental variance and reduce genetic variance.(28)

The significance of *A*, *C*, and *D* was tested by removing them sequentially in specific submodels, eventually leading to a model that gives the most parsimonious fit to the data. This leads to a model explaining the variance and covariances with as few parameters as possible. Submodels were compared with the full model by using a chi-square test. From the best-fitting model, it is possible to estimate the proportion of total variance attributable to *A*, *D*, *C*, and *E*. Trivariate Cholesky decomposition parameterization(27) was calculated in order to examine genetic and environmental contributions to the covariances among body composition (LM, FM) and BMD. This provides estimates of the genetic correlation (*r*_*g*_), the common environmental correlation (*r*_*c*_), and the unique environmental correlation (*r*_*e*_) between a pair of measures. For example, the genetic correlation indicates the extent to which genetic effects on one trait correlate with genetic effects on another trait independent of the heritability of the two traits. A genetic correlation of 1.0 would indicate that genetic influences on the two traits are identical, whereas a genetic correlation of 0 would indicate that completely different genes influence the two traits. By incorporating the heritability of the measures, it is also possible to estimate the extent to which genetic factors contribute to the observed phenotypic correlation between the traits. To study the effects of the EDAC selection on the twin model estimates,(29) we fitted univariate models for self-reported BMI using data from the full FinnTwin16 sample (1532 monozygotic and 3247 dizygotic twin individuals) and compared the estimates with measured BMI from the selected subsample. The heritability estimate from the best-fitting *AE* models of self-reported BMI in the full sample [*A* = 0.77, 95% confidence interval (CI) 0.74–0.80] was not significantly different from the heritability estimate of measured BMI in the selected sample (*A* = 0.72, 95% CI 0.58–0.81), as judged by the 95% CIs. All quantitative genetic model fitting was carried out with the software package MX (6th edition, Richmond, VA, USA).(27,30)

## Results

### Clinical characteristics of the sample

#### Men and women

This study sample consisted of 154 men and 147 women. Men and women were similar with respect to BMI (25.3 ± 3.8 kg/m^2^ versus 24.3 ± 5.1 kg/m^2^, *p* = .13), but men were significantly heavier (80.6 ± 13.4 kg versus 66.9 ± 13.8 kg, *p* < .001), taller (178.5 ± 5.7 cm versus 166.0 ± 5.7 cm, *p* < .001) and had significantly higher LM (58.2 ± 7.4 g versus 40.3 ± 4.2 g, *p* < .001), whole-body bone mineral content (BMC; 3231 ± 429 g versus 2543 ± 316 g, *p* < .001), and whole-body BMD (1.27 ± 0.10 g/cm^2^ versus 1.17 ± 0.10 g/cm^2^, *p* < .001) than women. Men had significantly lower FM (19.5 ± 9.3 kg versus 23.8 ± 11.3 kg, *p* = .002) and body fat percentage (23.1% ± 8.5% versus 34.1% ± 9.5%, *p* < .001) compared with women.

#### Monozygotic and dizygotic twins

A total of 115 monozygotic (57 full pairs, 1 individual) and 186 same-sex dizygotic (92 full pairs, 2 individuals) twin individuals were studied (Table 1). No significant differences were observed between the monozygotic and dizygotic twins in the means of age, anthropometrics, and bone parameters (Table 1). Therefore, the assumption of the twin method that the trait means do not differ between monozygotic and dizygotic twins was fulfilled. The difference in BMI between the heavier and leaner cotwins ranged from 0.01 to 10.2 kg/m^2^ (mean ± SD: 2.6 ± 2.4 kg/m^2^) in monozygotic pairs and from 0.1 to 15.2 kg/m^2^ (mean ± SD: 4.6 ± 3.6 kg/m^2^) in dizygotic pairs.

**Table 1:** Clinical Characteristics of the Sample

	Monozygotic twin individuals	Same-sex dizygotic twin individuals	*p* Value
Number of twin individuals	115	186	
Age (years)	27.1 ± 1.9	27.7 ± 2.1	.08
Weight (kg)	74.2 ± 14.4	73.8 ± 15.8	.86
Height (cm)	171.5 ± 8.3	172.9 ± 8.6	.30
BMI (kg/m^2^)	25.2 ± 4.5	24.6 ± 4.5	.35
Body fat (%)	29.1 ± 10.2	28.1 ± 10.8	.53
Fat mass (kg)	22.0 ± 10.1	21.4 ± 10.8	.67
Lean mass (kg)	49.5 ± 10.8	49.4 ± 10.8	.97
Whole-body BMC (g)	2869 ± 498	2911 ± 520	.60
Whole-body BMD (g/m^2^)	1.22 ± 0.10	1.22 ± 0.10	.58
Head BMD (g/m^2^)	2.33 ± 0.28	2.28 ± 0.26	.27
Arm BMD (g/m^2^)	0.95 ± 0.12	0.97 ± 0.13	.34
Rib BMD (g/m^2^)	0.69 ± 0.07	0.70 ± 0.08	.72
Leg BMD (g/m^2^)	1.34 ± 0.14	1.35 ± 0.14	.63
Pelvis BMD (g/m^2^)	1.19 ± 0.13	1.21 ± 0.14	.33
Spine BMD (g/m^2^)	1.06 ± 0.11	1.07 ± 0.12	.59

Data are mean ± SD. *p* Value is calculated using the Wald test for equality of means in monozygotic and dizygotic twins. BMI = body mass index; BMC = bone mineral content; BMD = bone mineral density.

### Relationship between soft tissue body composition and BMD in men and women

In the whole sample, phenotypic correlations between BMD and LM and BMD and FM were significant at all sites (Table 2). To examine possible gender difference, analyses of the trait relationships also were conducted separately by gender. BMD at the head, ribs and spine correlated better with FM than with LM, whereas BMD at the arms, legs, pelvis, and whole body correlated better with LM than with FM in both genders (Table 2). Multiple regression analyses adjusted for height indicated that the relationship between soft tissue composition and BMD was similar in men and women. In both genders, LM was independently associated with BMD at all regional sites, except at the head. FM was independently associated with BMD at the ribs, pelvis, and spine in both genders and with head BMD in men (data not shown). LM was independently associated with whole-body BMD in men (β = 0.0066 ± 0.0011, *p* < .001) and women (β = 0.0076 ± 0.0017, *p* < .001). FM was independently associated with whole-body BMD (β = 0.0027 ± 0.0007, *p* < .001, for men and β = 0.0012 ± 0.0007, *p* = .07, for women), although the association was only marginally significant in women.

**Table 2:** Phenotypic Partial Correlations Between Soft Tissue Body Composition and Bone Mineral Density (BMD) in Men, Women, and the Whole Sample (Men and Women Combined)

	Men (*n* = 154)^a^		Women (*n* = 147)^a^		Whole sample (*n* = 301)^b^	
			
BMD region	Lean mass	Fat mass	Lean mass	Fat mass	Lean mass	Fat mass
Head	0.18*	0.32***	0.07	0.15	0.14*	0.26***
Arms	0.49***	0.20*	0.43***	0.18	0.45***	0.21**
Ribs	0.50***	0.54***	0.50***	0.52***	0.50***	0.54***
Legs	0.39***	0.19*	0.36***	0.14	0.38***	0.18*
Pelvis	0.46***	0.39***	0.47***	0.39***	0.46***	0.41***
Spine	0.37***	0.43***	0.35***	0.46***	0.37***	0.45***
Whole body	0.51***	0.38***	0.43***	0.24*	0.47***	0.34***

a Data are adjusted for height.

b Data are adjusted for height and gender.

* *p* < .001;

** *p* < .01;

*** *p* < .05.

### Relationship between soft tissue body composition and BMD independent of genetic effects

**Fig. 1:**
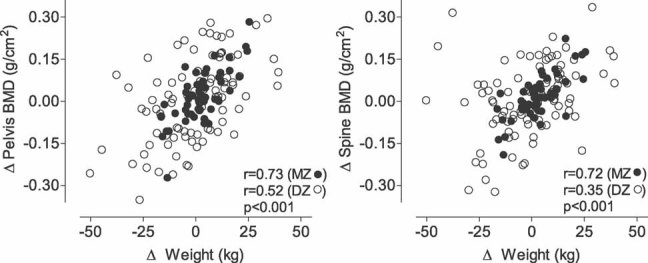
Height-adjusted Pearson correlations between intrapair differences in body weight and intrapair differences in BMD at the pelvis and spine in 57 monozygotic and 92 same-sex dizygotic twin pairs. BMD = bone mineral density; Δ = intrapair difference.

The strongest height-adjusted correlations between intrapair differences in body weight and intrapair differences in BMD were seen at the pelvis and spine (Fig. 1). The correlations were higher in monozygotic than in dizygotic pairs, indicating that acquired body weight is strongly associated with BMD independent of genetic influences. In monozygotic pairs, height-adjusted partial correlations between intrapair differences in BMD and intrapair differences in soft tissue body composition were stronger with FM (*r* = 0.41–0.66, *p* < .01) than with LM (*r* = 0.22–0.48, *p* < .05) with the exception of arm BMD, which was only significantly correlated with LM (*r* = 0.36, *p* < .01). The opposite was true for dizygotic pairs, where intrapair correlations were higher with LM (*r* = 0.35–0.54, *p* < .001) than with FM (*r* = 0.25–0.44, *p* < .05) at all sites, with the exception of rib BMD (FM: *r* = 0.59, *p* < .001; LM: *r =* 0.54, *p* < .001) and head BMD, which was only significantly correlated with FM (*r* = 0.21, *p* < .05).

Table 3 shows the results of the multiple linear regression analyses with adjustment for gender and height performed to identify the independent associations between soft tissue composition and BMD. At most sites, intrapair differences in both FM and LM were independently associated with the intrapair difference in BMD in both zygosity groups, demonstrating a significant relationship with both soft tissue compartments after controlling for genetic influences. However, the intrapair difference in LM was a significantly stronger predictor of whole-body BMD than the intrapair difference in FM in both monozygotic and dizygotic pairs. The proportion of variance of intrapair differences in whole-body BMD attributable to intrapair differences in soft tissue composition was much lower in dizygotic (41%) than in monozygotic (65%) twins.

**Table 3:** Regression Coefficient (β ± SE) for the Relationship Between Intrapair Differences (Δ) in Soft Tissue Composition (Lean Mass and Fat Mass) and Intrapair Differences in Bone Mineral Density (10^−2^ g/cm^2^) at Six Skeletal Sites and the Whole Body in 57 Monozygotic and 92 Same-Sex Dizygotic Twin Pairs

	Head	Arms	Ribs	Legs	Pelvis	Spine	Whole body
Model 1							
Monozygotic pairs							
Δ Lean mass (kg)	0.84 ± 0.41*	0.61 ± 0.26*	0.71 ± 0.16***	0.54 ± 0.18**	1.42 ± 0.34***	1.11 ± 0.29***	0.84 ± 0.15***
*r*^2^	0.11	0.10	0.34	0.32	0.34	0.29	0.44
Dizygotic pairs							
Δ Lean mass (kg)	0.14 ± 0.38	0.87 ± 0.17***	0.53 ± 0.10***	0.82 ± 0.15 ***	1.12 ± 0.19***	0.58 ± 0.19**	0.80 ± 0.12***
*r*^2^	0.02	0.25	0.29	0.27	0.32	0.14	0.34
Model 2							
Monozygotic pairs							
Δ Fat mass (kg)	0.51 ± 0.15**	0.09 ± 0.10	0.31 ± 0.06***	0.21 ± 0.07**	0.72 ± 0.11***	0.61 ± 0.10***	0.32 ± 0.06***
*r*^2^	0.21	0.02	0.43	0.32	0.50	0.49	0.44
Dizygotic pairs							
Δ Fat mass (kg)	0.37 ± 0.18*	0.38 ± 0.09	0.32 ± 0.04***	0.20 ± 0.08*	0.45 ± 0.10***	0.32 ± 0.09**	0.31 ± 0.07***
*r*^2^	0.06	0.21	0.40	0.08	0.20	0.16	0.21
Model 3							
Monozygotic pairs							
Δ Lean mass (kg)	0.57 ± 0.39	0.58 ± 0.27*	0.55 ± 0.13***	0.44 ± 0.17*	1.04 ± 0.27***	0.79 0.23**	0.68 ± 0.12***
Δ Fat mass (kg)	0.46 ± 0.15**	0.05 ± 0.10	0.27 ± 0.05***	0.17 ± 0.07*	0.63 ± 0.10***	0.54 ± 0.09***	0.26 ± 0.05***,a
*r*^2^	0.24	0.10	0.58	0.40	0.61	0.58	0.65
Dizygotic pairs							
Δ Lean mass (kg)	-0.11 ± 0.40	0.69 ± 0.18***	0.26 ± 0.04***	0.77 ± 0.16***	0.98 ± 0.19***	0.43 ± 0.19*	0.67 ± 0.12***
Δ Fat mass (kg)	0.39 ± 0.20	0.27 ± 0.09**	0.36 ± 0.09***	0.08 ± 0.08^b^	0.29 ± 0.09**,a	0.25 ± 0.10*	0.20 ± 0.06**,a
*r*^2^	0.06	0.33	0.50	0.28	0.37	0.20	0.41

*Note:* All models were adjusted for height and gender.

* *p* < .001;

** *p* < .01;

*** *p* < .05.

a Significantly different from the regression coefficient for lean mass at *p* < .01.

b Significantly different from the regression coefficient for lean mass at *p* < .001.

### Intraclass correlations and heritability estimates

Intraclass correlations and heritability estimates of bone parameters were calculated using the combined data for men and women and adjusted for gender and height. The intraclass correlations for BMD were higher among monozygotic twins than among dizygotic twins at all sites (Table 4), further indicating genetic influences, which were confirmed subsequently by model fitting. For BMD variables, the *AE* model fitted the data better than the *ACE* and *ADE* models. The *AE* model also was superior to the *E* model. Hence the most appropriate model contained only additive genetic and unique environmental components (*AE* model). For LM and FM, the *ADE* model fitted the data slightly better than the *AE* model (chi-square change *p* = .023 for FM and .002 for LM). In the *ADE* model, all the genetic influence was placed on the *D* effect. Since a model in which all the variance is due to dominance and none is additive is biologically implausible, *AE* models were used in the subsequent analyses. Univariate analysis confirmed the presence of a genetic influence at each of the six skeletal sites and the whole body (Table 4). Genetic factors explained 81% (95% CI 70%–88%) of the variation in LM and 61% (95% CI 41%–75%) of the variation in FM.

**Table 4:** Intraclass Correlations and Heritability Estimates of Bone Mineral Density (BMD) Adjusted for Gender and Height at Six Skeletal Sites and the Whole Body in 57 Monozygotic (MZ) and 92 Same-Sex Dizygotic (DZ) Twin Pairs

BMD region	MZ correlation coefficient (95% CI)	DZ correlation coefficient (95% CI)	Heritability estimate (95% CI)
Head	0.94 (0.91–0.97)	0.49 (0.33–0.65)	0.93 (0.89–0.95)
Arms	0.80 (0.71–0.89)	0.26 (0.07–0.45)	0.80 (0.69–0.87)
Ribs	0.70 (0.56–0.83)	0.35 (0.17–0.53)	0.74 (0.61–0.83)
Legs	0.91 (0.86–0.95)	0.42 (0.26–0.59)	0.91 (0.86–0.94)
Pelvis	0.68 (0.55–0.82)	0.36 (0.18–0.54)	0.72 (0.58–0.81)
Spine	0.71 (0.58–0.84)	0.35 (0.17–0.53)	0.74 (0.61–0.83)
Whole body	0.86 (0.80–0.93)	0.39 (0.22–0.57)	0.87 (0.80–0.91)

*Note:* Heritability estimates are from univariate models.

### Genetic and environmental contributions to the covariance among LM, FM, and BMD

**Fig. 2:**
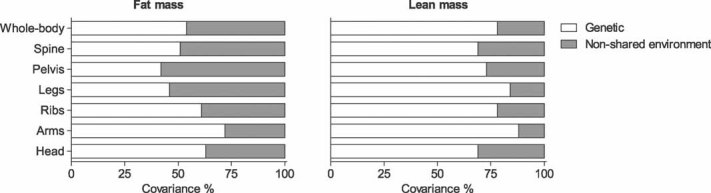
Proportion of covariance between lean mass and BMD and fat mass and BMD at six skeletal sites and the whole body accounted for by each of the variance components from a trivariate genetic model adjusted for gender and height (*n* = 57 monozygotic and 92 same-sex dizygotic twin pairs).

The covariance between FM and BMD was mediated evenly by genetic and environmental influences (Fig. 2). For example, genetic and environmental influences that are shared by FM and whole-body BMD accounted for 54% and 46% of the phenotypic correlation between these traits, respectively. The covariance between LM and the different BMD sites was explained in large part by genes (covariances ranged from 69% to 88%) and to a lesser degree by the environment (12% to 31%; Fig. 2). Constraining the genetic and environmental covariances for LM and FM to be equal gave a significantly worse fit at the legs (*p* < .05), pelvis (*p* < .001), head (*p* < .001), ribs (*p* < .001), spine (*p* < .001) and whole body (*p* < .01), indicating that genetic influences contribute to a larger proportion of the covariance between LM and BMD than between FM and BMD. The extent to which two traits share the same genetic and unique environmental effects is given in Table 5. The genetic correlations between LM and BMD (*r*_*g*_ = 0.46 for the whole body) were greater than those between FM and BMD (*r*_*g*_ = 0.25 for the whole body) at most skeletal sites (Table 5). These correlations also emphasize the importance of unique genetic factors for each trait. There also was some overlap of both genetic (*r*_*g*_ = 0.29, 95% CI 0.06–0.47) and unique environmental factors (*r*_*e*_ = 0.31, 95% CI 0.04–0.54) that influence FM and LM.

**Table 5:** Genetic and Environmental Correlations Between Lean Mass and Bone Mineral Density (BMD) and Fat Mass and BMD at Six Skeletal Sites and the Whole Body in 57 Monozygotic and 92 Same-Sex Dizygotic Twin Pairs

	Genetic correlations		Environmental correlations	
		
BMD region	Lean mass^a^	Fat mass^b^	Lean mass^c^	Fat mass^d^
Head	0.08 (–0.06–0.23)	0.18 (0.01–0.34)	0.28 (0.01–0.51)	0.47 (0.23–0.66)
Arms	0.54 (0.39–0.67)	0.26 (0.03–0.44)	0.29 (0.01–0.53)	0.26 (0.00–0.49)
Ribs	0.50 (0.34–0.63)	0.50 (0.30–0.64)	0.55 (0.32–0.71)	0.67 (0.49–0.79)
Legs	0.39 (0.25–0.51)	0.11 (–0.09–0.28)	0.46 (0.21–0.65)	0.48 (0.24–0.66)
Pelvis	0.45 (0.28–0.58)	0.23 (–0.01–0.42)	0.56 (0.35–0.71)	0.68 (0.50–0.80)
Spine	0.29 (0.11–0.45)	0.29 (0.08–0.47)	0.49 (0.26–0.67)	0.65 (0.46–0.78)
Whole body	0.46 (0.32–0.58)	0.25 (0.05–0.42)	0.65 (0.46–0.78)	0.68 (0.50–0.80)

*Note:* Correlations are from a trivariate genetic model adjusted for gender and height.

a Correlation between genetic variance components of lean mass and BMD.

b Correlation between genetic variance components of fat mass and BMD.

c Correlation between unique environmental variance components of lean mass and BMD.

d Correlation between unique environmental variance components of fat mass and BMD.

## Discussion

In this study we evaluated the relationship of soft tissue body composition with BMD at the whole body and six skeletal sites in healthy twins who have largely reached the age of peak bone mass.(31) Given the fact that a high peak bone mass attained during young adulthood decreases the risk of osteoporosis and fractures later in life,(32) it is important to identify the factors that determine BMD in this period of life.

Our finding of a positive association between body weight and BMD is in line with a number of previous studies that have evaluated this association in singletons.(3–5) The results of our intrapair analysis in monozygotic twin pairs confirm and extend previous reports by showing that this association remains when genetic factors are controlled for. However, body weight is composed of FM and LM, and the relative importance of these two components to BMD is less clear and widely debated.(4,8–11,15,21,33) This study provides further insight into this question, suggesting that an association exists between both soft tissue compartments and BMD at most skeletal sites after controlling for potential confounding genetic influences. It is noteworthy that at the whole-body level, LM was a significantly better predictor of BMD than FM. An intrapair difference of 1 kg in LM was associated with a difference of 0.0068 g/cm^2^ in whole-body BMD, whereas an intrapair difference of 1 kg in FM was associated with a difference of 0.0026 g/cm^2^ in whole-body BMD in monozygotic twin pairs. This finding is in line with one earlier twin study by Nguyen and colleagues,(15) who showed that both LM and FM are independently associated with whole-body BMD in 20- to 83-year-old female twins. Since lean mass (a surrogate for skeletal muscle mass) is related to physical activity(34) and certain dietary patterns,(35) our results are clinically relevant because they demonstrate that lifestyle modifications aimed at increasing physical activity levels and improving eating habits could be useful in reducing the risk of osteoporosis and obesity simultaneously.

The positive association between body weight and BMD has been explained by a combination of mechanical and hormonal factors.(12) Only few studies have addressed the possibility that genetic factors could contribute to the positive associations between LM and BMD or between FM and BMD.(15,36) In this study, the genetic correlation between LM and whole-body BMD was estimated to be 0.44, which means that genetic factors influencing these two tissues show moderate overlap. The genetic correlation between FM and BMD was lower (*r*_*g*_ = 0.25) than that for LM at most sites, indicating that LM and BMD share more genes in common than do FM and BMD. Recently, bivariate genome linkage analyses were performed to explore shared genomic regions for BMD and body composition traits (eg, LM(37) and FM(38)). For example, in a bivariate linkage study for LM and BMD conducted in a large sample of 4498 individuals, 7p22 emerged as an interesting chromosome region with pleiotropic effects on total-body LM and spine BMD. In addition, 15q13 was found to be an important candidate chromosome region commonly influencing total-body LM and spine BMD in women. Potential candidate genes that are relevant to both LM and BMD in these regions include *TWIST* (*twist homologue 1*), *IL6* (*interleukin 6*), and *GREM1* (*gremlin 1*).(37) In line with the significant genetic correlation between LM and FM (*r*_*g*_ = 0.29) in this study, a bivariate linkage analysis also suggests several genomic regions (20p12, 3p26-25, and Xp22) that may jointly influence FM and LM.(39) The results of this study differ from those of Nguyen and colleagues,(15) who examined the relationships among LM, FM, and BMD in Australian female twins. In their study, the genetic correlation between LM and whole-body BMD was 0.09 and nonsignificant. In our study, the associations between the two soft tissue compartments and BMD were mediated via genetic and unique environmental factors, whereas in the study by Nguyen and colleagues, these associations were mediated mainly via common environmental influences.(15) The Australian sample differs from our sample in several aspects because it was more heterogeneous with regard to age range (20 to 83 years), included only female twin pairs, and zygosity was determined by self-report.

As anticipated, whole-body BMD was substantially heritable, with 86% of the total variance accounted for by genetic effects in young adults. Many candidate genes have been proposed as being involved in regulating BMD, and the most intensively studied include the *vitamin D receptor* (*VDR*) gene, the *collagen type Iα1* gene (*COLIA1*), and the *estrogen receptor α* (*ERα*) gene.(40) In this study, we also showed that peak BMD is determined by body weight and that both LM and FM contribute to BMD in young adulthood. Lifestyle factors such as physical activity and smoking also have been documented to contribute to the level of achieved peak BMD.(41) An increasing body of literature suggests that gene-environment interactions may be important in determining BMD. Suuriniemi and colleagues showed that the effect of the *ERα* polymorphism on loaded bone sites in 10- to 13-year-old girls varies according to their leisure-time physical activity level.(42) Similarly, a polymorphism in the *catechol-O-methyltransferase* (*COMT*) gene was found to modulate the association between physical activity and peak BMD in young men.(43)

Some strengths and limitations of this study should be considered when interpreting our findings. Limitations of the study include the cross-sectional design and the inability to extrapolate our findings to other ethnic groups, children, or older adults. The strengths of our study include the use of a genetically informative sample of twins with a wide range of intrapair differences in BMI, simultaneous examination of the effects of acquired body weight and the effects of genes on BMD, the narrow age range, the entire limitation to young adults, and the use of measured rather than self-reported height and weight. Moreover, we used DXA, which is considered the “gold standard” technology for measuring BMD because it is the most extensively validated test for predicting fracture outcomes.(44) This technique has been shown to measure bone mass precisely and accurately.(23,24) However, the method is also associated with some notable disadvantages. The DXA instrument provides an estimate of density expressed as grams per projected area (areal BMD, in g/cm^2^). This estimate is not a measure of volumetric density (g/cm^3^) because it provides no information about bone depth. Therefore, DXA tends to overestimate the BMD of taller subjects and underestimate the BMD of smaller subjects.(45). Another limitation of the DXA technique is that extremely obese subjects frequently exceed the tested weight limits and in some cases do not physically fit within the scanning area.(46) Despite these limitations, DXA is the most widely used method to estimate body composition both in research studies and in clinical practice.

The sample used in this study was selected on the basis of pairwise discordance and concordance for obesity, as assessed by BMI calculated from self-reported weight and height. This selection procedure yielded a highly informative sample for studying the relationship between body composition and BMD. Concerning the representativeness of the twin model estimates from this selected sample, it has been shown with simulated twin data that the bias resulting from the EDAC selection is minimal.(29) Because data selected with the EDAC procedure are technically “missing at random,” unbiased model estimates are in fact expected on the basis of missing-data theory.(47) In this study, the heritability of BMI in the subsample was very close to that of the full sample and in agreement with heritability estimates derived from earlier twin studies on young adults.(14)

In conclusion, our data provide evidence that the association between soft tissue composition and BMD is mediated by genetic and unique environmental factors. The higher genetic correlation between LM and BMD than between FM and BMD at various skeletal sites indicates that BMD may have more genes in common with LM than with FM. The association between both soft tissue compartments and BMD persisted in intrapair analyses in monozygotic twins, underscoring the importance of acquired body weight on BMD independent of genetic influences. Furthermore, we found that LM is a significantly better predictor of whole-body BMD than FM per kilogram of tissue mass in young adulthood when genetic factors are controlled for. Importantly, these results address earlier uncertainties concerning the association between soft tissue body composition and BMD and highlight the need to search for underlying genetic as well as biological mechanisms.
